# *Vayu*: An Open-Source Toolbox for Visualization and Analysis of Crowd-Sourced Sensor Data

**DOI:** 10.3390/s21227726

**Published:** 2021-11-20

**Authors:** Sachit Mahajan

**Affiliations:** Computational Social Science, Department of Humanities, Social and Political Sciences, ETH Zürich, 8092 Zürich, Switzerland; sachit.mahajan@gess.ethz.ch

**Keywords:** open-source, air quality, data analysis, citizen data

## Abstract

Recent advances in sensor technology and the availability of low-cost and low-power sensors have changed the air quality monitoring paradigm. These sensors are being widely used by scientists and citizens for monitoring air quality at finer spatial-temporal resolution. Such practices are opening up opportunities to enhance the traditional monitoring networks, but at the same time, these sensors are producing large data sets that can become overwhelming and challenging when it comes to the scientific tools and skills required to analyze the data. To address this challenge, an open-source, robust, and cross-platform sensor data analysis toolbox called *Vayu* is developed that allows researchers and citizens to do detailed and reproducible analyses of air quality data. *Vayu* combines the power of visualization and statistical analysis using a simple and intuitive graphical user interface. Additionally, it offers a comprehensive set of tools for systematic analysis such as data conversion, interpolation, aggregation, and prediction. Even though *Vayu* was developed with air quality research in mind, it can be used to analyze different kinds of time-series data.

## 1. Introduction

In recent years, the air quality monitoring paradigm has rapidly changed due to the use of the Internet of Things (IoT) and participatory sensing methods [1]. These developments have allowed researchers to use low-cost sensing solutions to monitor the environment at a finer spatial and temporal scale. To complement the official air quality monitoring systems, citizen-driven air quality monitoring networks have also been created all over the world. These networks are mainly run by Citizen Scientists who are community members involved in scientific research [2,3]. As citizen science grows bigger, more ambitious projects are undertaken by the scientists in collaboration with the citizens [4]. To promote inclusiveness, transparency, and open data, researchers have been developing co-creation studies where citizens are actively involved in large-scale deployment of low-cost air quality sensors to create air quality maps and tools not just for one city, but for an entire region [5]. This has led to an increase in the availability of air quality data as well as interest from multiple stakeholders who want to explore the real-time data to extract locally relevant information [6]. However, processing, aggregation, analysis, and visualization of low-cost sensor data is a formidable task that requires a comprehensive computational framework and expert knowledge of different programming languages and data analysis tools. In most air quality participatory sensing studies, citizen scientists, and other stakeholders depend on computational researchers to process and analyze the data to unravel meaningful insights. In many cases, commercial packages like SPSS Statistics [7] from IBM, Statistica from Statsoft [8], or open-source professional software like R Studio are used [9]. Most of the citizen-driven environment monitoring networks have dedicated applications and platforms for data visualization and analysis. For example, Purple Air PA-II sensors have a map-based visualization and the AirSensor R package [10]; AirBox has chatbot [11], Grafana based visualization, pollution maps, route finding application [5]; Luftdaten has a network map [12] and AirBeam has AirCasting map [13]. However, all these applications and resources are sensor-specific. So, for analyzing data from different sensor networks, the user would have to do it separately on a sensor-specific platform. In addition to these sensor-specific platforms, there are several R packages like OpenAir [14] and PWFSLSmoke [15] that can be used for visualizing and analyzing air quality data. But to work with these packages, users would need basic programming skills. Despite the availability of sensor-specific tools and programming libraries, exploration, statistical analysis, and visualization of sensor data sets can still be challenging for citizen scientists and the majority of researchers who don’t have training in programming and data science. Also, many of the commercial applications are black-box algorithms, which is problematic in terms of transparency and open science.

One way to address these challenges is to create appropriate and open-source tools that can reduce technical barriers especially related to programming, and allow experts as well as non-experts to analyze and interpret data in a meaningful way [16]. In this work, an open-source toolbox *Vayu* is presented that enables researchers as well as citizen scientists independent of their computational skills to analyze their air quality data. *Vayu* is a Sanskrit word that means “air” in English. The current version of *Vayu* addresses the challenges and concerns mentioned above by (i) Creating an open, free, and ready to use a toolbox that can process data from different air quality sensor networks, as well as complex time-series data from other sources, (ii) Providing an intuitive graphical user interface (GUI) that allows users to easily visualize and analyze their data, (iii) Offering a variety of options to plot data (line plots, scatter plots and, bar plots) and statistically analyze the data using functions like data interpolation, summarization, aggregation, correlation, linear regression, and naïve Bayes classification to analyze complex data, and (iv) Facilitating easy export of charts, figures, and information-rich statistical analyses that can be used for reporting/publication with little post-processing. Furthermore, *Vayu* is distributed as an open-source toolbox that allows advanced users to create custom functions and add new features to enhance the toolbox.

In what follows, I first present the system architecture of *Vayu*. That is followed by a detailed description of the analysis workflow where *Vayu* is tested with different air quality sensor data sets. I subsequently present the results of a series of tests that were performed to understand how different statistical analysis methods work. The features of *Vayu* are then compared with existing state-of-the-art air quality sensor data analysis tools and libraries. The final section discusses some future directions.

## 2. Methods

### 2.1. Toolbox Design

*Vayu* is a Python (>3.7) based toolbox that takes advantage of well documented Python packages. The reason behind choosing Python for writing this toolbox was the availability of a wide range of open-source data analysis libraries, and user-specific code adjustments that can be achieved by researchers from different backgrounds. *Vayu* is an open-source toolbox and relies on the following Python modules: (i) PyQt5 [17]—set of Python bindings for C++ widget library Qt v5 are used to build the GUI. (ii) Pandas [18]—library for data manipulation and analysis. (iii) Scikit-learn [19]—library to implement machine learning in Python. (iv) Matplotlib [20]—library for generating plots that are embedded in PyQt widgets.

**Figure 1:**
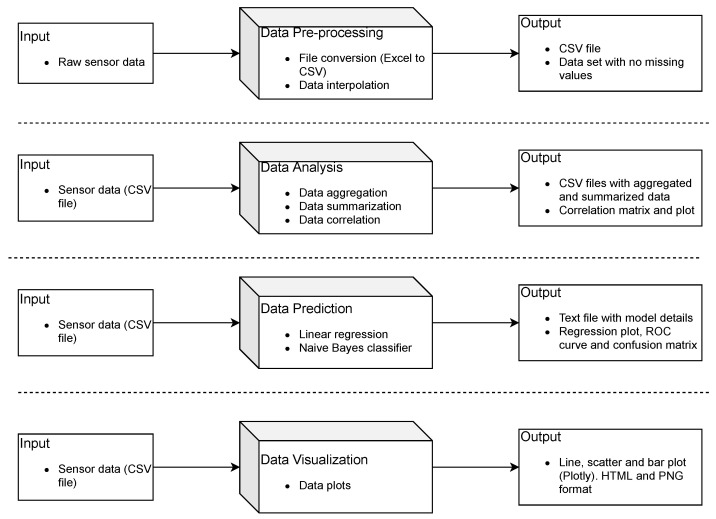
*Vayu* workflows: The toolbox supports the analysis of sensor data by including four workflows. The toolbox accepts external inputs for different workflows and produces output that is stored locally.

All these four modules are well documented and actively used by the open-source developer community. The GUI built with PyQt5 puts together different windows, widgets, and elements of the toolbox. While designing this toolbox, the main objective was to develop an intuitive application that would facilitate usability for people with different skill levels. Different levels of interaction with the data allow the user to uncover meaningful insights without using coding or any query language. Figure 1 gives an overview of the different workflows of *Vayu*.

### 2.2. Reporting, Maintenance and Community-Driven Development

As this is an open-source toolbox, users would be encouraged to report issues and unexpected behaviour via GitHub [21].

### 2.3. Example Data

Several example data sets have been included in the dataset folder of the GitHub repository [21]. This will facilitate an easy start for the users who want to use this toolbox. A fully documented and easy to follow step-by-step guide is also included that will allow users to perform data analysis using different data sets: (i) PurpleAir [22]: PurpleAir sensors network includes thousands of air quality monitors mainly deployed around North America. The data is regularly used by citizen scientists as well as local and regional environmental monitoring agencies. (ii) AirBox [23]: AirBox sensors are widely deployed around the world specifically in South-East Asia. At present, there are more than 20,000 plus devices deployed in 59 countries. The data is regularly used by scientists, citizens, and policymakers. (iii) Luftdaten [24]: Luftdaten is a citizen science project that runs a global network of air quality monitors to obtain fine-grained air quality data. (iv) A custom data set is also created and tested to show users how they can analyze custom data from IoT devices. The step-by-step guide will be extended regularly by adding example data from more sensor networks.

## 3. Results and Discussion

The idea behind the development of this toolbox is to simplify the process of analysis of air quality data. A simplified workflow is created for data pre-processing, analysis, prediction, and visualization. This allows the users to interpret the data, analyze the patterns, perform visual data inspection, as well as have reusable results for sharing within the research or citizen science community. The following paragraphs discuss the general aspects of the toolbox and give an overview of different features related to data organization and plotting, data analysis, and data prediction. The example data sets used for testing different features are available in the GitHub repository [21].

### 3.1. Data Organization and Plotting

The current version of the toolbox accepts data in different file types including comma-separated values (CSV) files and Excel. As most of the citizen science air quality monitoring projects allow you to download the data as a CSV file [5,22,24], it is set as the default file type the user should upload to perform different actions on the data. If the user has the data in Excel file format, the toolbox allows the user to convert it into CSV format for further analysis. The next step of data organization includes cleaning the data set by removing missing values. Low-cost sensors occasionally show missing data that is usually due to loss of transmission power or device failure [25]. From the quality control perspective, it is important to clean the missing data before the data analysis [26]. The *data interpolation* feature of the toolbox removes the missing data and uses linear interpolation to estimate the missing data. The linear interpolation method has been widely used for air quality data sets [27,28]. Linear interpolation estimates the missing values by fitting a straight line between the two data points. The missing values are calculated using the line equation [28].

(1) $$Y=Y_{1}+\frac{\left(X-X_{1}\right)}{\left(X_{2}-X_{1}\right)}\times \left(Y_{2}-Y_{1}\right)$$

In Equation (1), *X* is the known data point, *Y* is the value to be determined, $X_{1}$ and $Y_{1}$ are the coordinates that are below the known *X* value, and $X_{2}$ and $Y_{2}$ are the coordinates that are above the *x* value. The interpolated data is saved as a CSV file locally and can be used to initiate data visualization and analysis.

**Figure 2:**
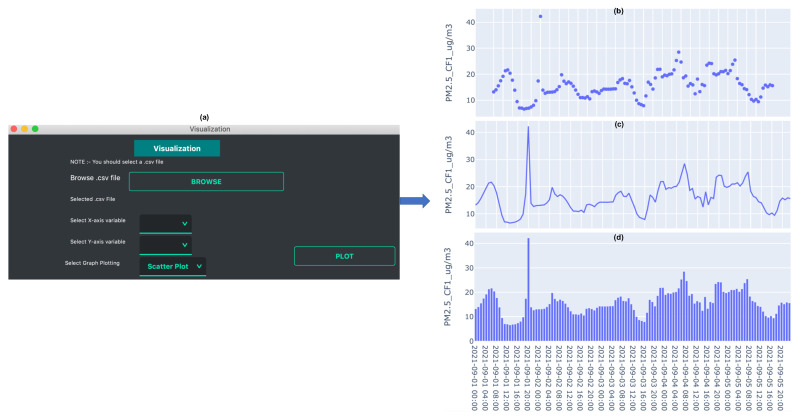
Data visualization example: (**a**) Snapshot of data visualization GUI, (**b**) scatter plot, (**c**) line plot, and (**d**) bar plot.

Plots can be created instantaneously by selecting the column headers in the X and Y axis boxes, and choosing the plot type (scatter, line and bar). For example, Figure 2 shows the output of the *data visualization* function. The data from “Tutorial_PurpleAir.csv” is used to create a scatter, bar, and line plot. The plots are generated using Plotly which is an interactive browser-based graphing library for Python [29]. The generated plots are saved in two file formats, .html and .png. This gives the user flexibility to use the plots for different purposes, for example, .html files can be easily shared and embedded within a web page and .png files can be used for reports, publications, etc.

### 3.2. Data Analysis

**Figure 3:**
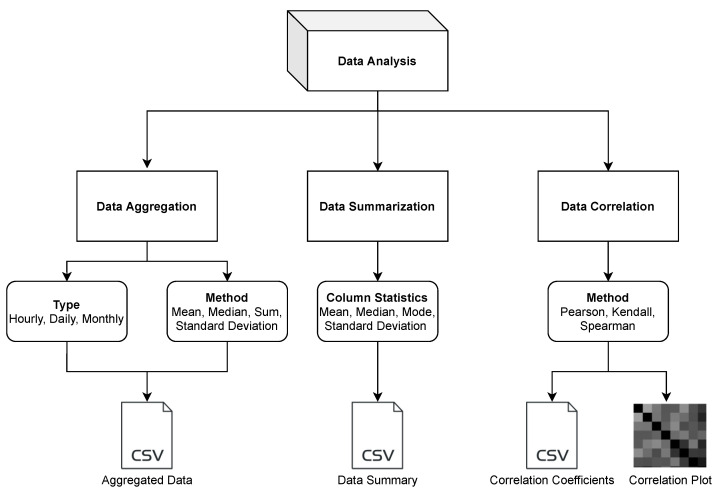
Data analysis workflow showing different functions, options, and outputs.

*Vayu* offers a choice of computational functions to analyze and explore sensor data. The functions can be applied to the columns in the data set. Figure 3 gives an overview of different features of the data analysis function. Data processing and statistical analysis are the key tasks of sensor data analysis. The aim of the data analysis workflow is to provide users with the options to recompile the data in the required format, understand the relationship between different variables in the data set, and provides a descriptive analysis of the data set.

**Figure 4:**
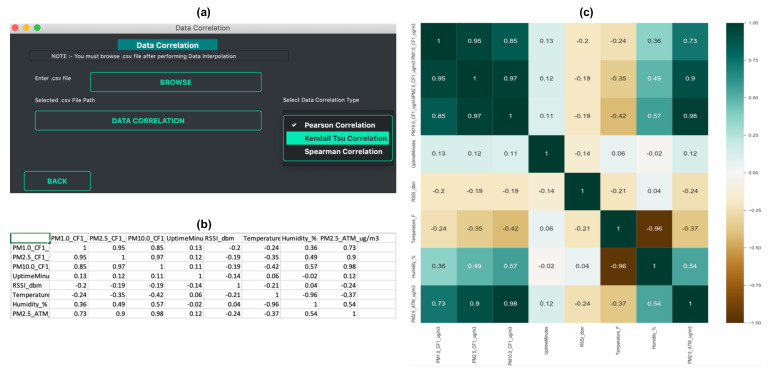
Data correlation example: (**a**) Snapshot of data correlation GUI, (**b**) output as a CSV file, and (**c**) correlation heatmap.

- Data Aggregation: Different sensors record measurements at different sampling frequencies. In many cases, either the data is too granular or not granular enough. Having an imbalanced time-series is a common problem and it often needs resampling solutions [30]. In scenarios such as comparing data sets from different sensors or comparing the data with regulatory monitors that have a different sampling frequency, the sensor data needs to be resampled. Also, in the case of doing a forecast at a different frequency, resampling may be required. Most of the widely used sensors have a sampling frequency in minutes [22,31]. In such cases, it is important to downsample the frequency, such as from minutes to hours, days, or months. The *data aggregation* function allows the user to downsample the data to hourly, daily, or monthly data. It uses the Pandas library to resample the data. A key requirement of using this function is that the data should have a DateTime type index. The users can use different methods like mean, median, sum, and standard deviation to perform data aggregation. The aggregated data is saved as a CSV file.
- Data Summarization: Data summarization is often needed to simplify the data interpretation and to understand the distribution of a variable within a data set. Some of the common ways to understand the data distribution are to look at the mean, mode, and median. These values are typically used to understand where the central part of the data is located. Another method is to look at the standard deviation, which acts as an index of variability. If the sensor data are widely scattered, the standard deviation would be larger, and if the data are clustered together, the standard deviation would be smaller. The *data summarization* function of the toolbox allows the user to look at the column statistics and download the result as a CSV file.
- Data Correlation: Looking at the correlation is an effective way to understand the relationship between different variables within a data set. For air quality data, it has been often observed that there is a correlation between air pollution concentration and meteorological factors. Air pollution is negatively correlated with humidity, wind speed, and precipitation, and positively correlated with atmospheric pressure [32]. Correlation allows the user to understand the strength of a relationship between two variables. Such information is useful when building models for calibration [33] or forecasting [34]. The *data correlation* function allows the user to perform data correlation. *Vayu* allows a user to calculate different correlation coefficients that are widely used in air quality research [35,36,37,38]: Pearson’s correlation, Kendall Tau’s Correlation, and Spearman correlation. Figure 4 shows the output of the *data correlation* function. The data from “Tutorial_PurpleAir.csv” is used to find the correlation coefficient. The output is a CSV file with correlation coefficients (Figure 4b) and a correlation heatmap (Figure 4c).

### 3.3. Data Prediction

*Vayu* promotes the visual interpretation of data, but it also offers several techniques to perform supervised learning. Supervised learning is a Machine Learning technique in which a model is trained on labeled data [39]. Such methods can be used for the prediction and classification problems.

**Figure 5:**
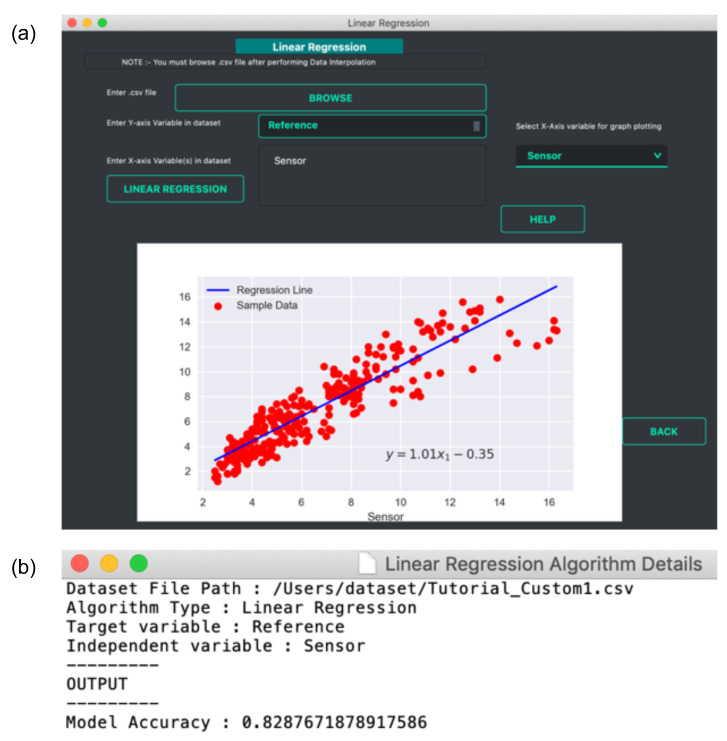
Linear regression example: (**a**) Snapshot of linear regression GUI with regression plot and (**b**) snapshot of the text file showing regression model details.

**Figure 6:**
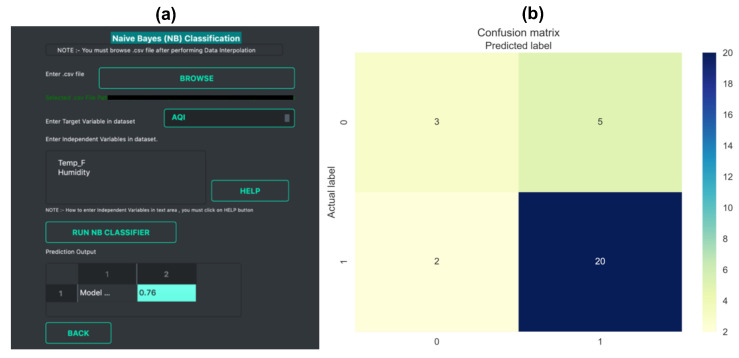
NB Classifier example with outputs: (**a**) Snapshot of NB classifier GUI and (**b**) confusion matrix.

**Figure 7:**
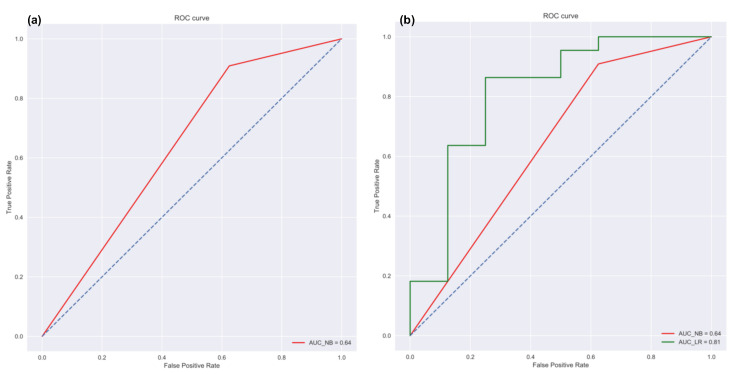
NB Classifier output: (**a**) ROC curve showing AUC for NB and (**b**) plot comparing AUC for NB and logistic regression.

- Linear Regression: The *data prediction* workflow allows the user to perform linear regression. A linear regression model finds the relationship between the independent and dependent variables. It is a relatively simple method but has been widely used for calibration studies [40] as well as for prediction tasks [41,42]. *Vayu* allows a user to use the linear regression function to build a model. It can be useful for citizen scientists as well as researchers who want to start with simple models to perform predictive analysis. Figure 5 shows an example of how the output of linear regression function looks. The data from “Tutorial_Custom1.csv” is used to build a regression model. This example data set contains two variables: data from a reference station, and data from a sensor. A linear regression model is developed to find the correction factor for the sensor. The GUI (as shown in Figure 5a) allows the user to upload the data, and select the dependent and independent variable. The output is a regression plot that shows the relationship between the independent and dependent variables. The plot is also saved as a .png file. A text file (as shown in Figure 5b) is also generated that shows the algorithm details and the model accuracy.
- Naïve Bayes Classifier: Methods like linear regression are efficient and useful when we are dealing with numeric data. But in some cases, the problem can be categorized as a classification problem. For example, a user wants to predict whether the Air Quality Index (AQI) would be “High” or “Low” based on different features. In such a case, a classification algorithm would have to be applied. *Vayu* toolbox allows the users to implement the Naive Bayes (NB) Classification algorithm to perform the classification tasks. NB is one of the most straightforward and fast classification algorithms [43], and is often used for air quality prediction [44]. NB is a supervised learning algorithm based on Bayes Theorem [45]. In simple words, generating a model using NB classifier includes creating the NB classifier, fitting the data set on the classifier, and performing prediction. The *data prediction* function allows the users to perform NB classification with binary labels. NB classification has several types and in the case of *Vayu*, Gaussian NB [46] is used. “Tutorial_Custom2.csv” has been used to test the method, and the results are shown in Figure 6 and Figure 7. The user can use the GUI to select the target variable and the independent variables (as shown in Figure 6a). The data is split between train and test set. The default setting has to be kept to 75% of data for training and the remaining 25% data for testing. This is followed by generating a model. The model is then evaluated by checking the accuracy of the model. The results are in the form of model accuracy, confusion matrix (Figure 6), and a receiver operating characteristic (ROC) curve (Figure 7). The confusion matrix summarizes the performance of the classification model on the test data. The ROC curve is created by plotting the true positive rate against the false-positive rate. The ROC curve shows the area under the curve (AUC) that provides an aggregate measure of performance. The output also includes an ROC curve (Figure 7b) that compares the performance of Gaussian NB to Logistic Regression [47]. This provides a user with an additional way of understanding the performance of different models. The ROC curves and the confusion matrix are saved as .png files, and the algorithm details are saved as a text file.

### 3.4. Comparison with Existing Tools

Table 1 compares *Vayu* with other air quality sensors analysis toolkits and software. Each toolkit and software has its strengths and weaknesses as they were designed for different user groups. The issue with sensor-specific software is that they might not work well in case the data from a different sensor is in a different format. With open-source libraries (for example, R packages), the user needs to be experienced in programming to analyze the data. *Vayu* provides a combination of features in one application that works for different sensor data as well as allows users from different backgrounds to analyze and visualize data without any need for programming. The open-source nature of the toolbox allows users with training in programming to add more functionalities as well as improve the existing features.

## 4. Conclusions and Future Directions

With citizen science and community-driven air quality monitoring becoming a common practice around the globe, it is important to find user-friendly and intuitive ways to analyze massive streams of sensor data. *Vayu* aims to support easy analysis and interpretation of air quality sensor data. Developed as a desktop-based, and non-sensor-specific data analysis and visualization toolbox, it equips the users to perform fast and comprehensive analysis of air quality data sets. The open-source nature of the proposed toolbox allows the developer community to build on the existing framework, collaborate, and add to and improve the features of *Vayu*.

The work discussed in the paper focused on the initial version of *Vayu* and explained its data processing, analysis, and visualization capabilities. Future work will include adding more functionalities related to outlier detection and time-series forecasting. Additional enhancements may include improvement in the user interface.

## Figures and Tables

**Table 1:** Comparison of *Vayu* with other tools.

Name	Open Source	Sensor Specific	GUI	Programming Requirement
AirSensor [10]	Yes	Yes	-	Yes
OpenAir [14]	Yes	No	-	Yes
DataViewer [10]	Yes	Yes	Web-based	No
PWFSLSmoke [15]	Yes	No	-	Yes
*Vayu*	Yes	No	Desktop App	No

## Data Availability

The source code of *Vayu* and all the data sets that are used in this paper are available on GitHub (https://github.com/sachit27/VAYU (accessed on 16 October 2021)).
